# Asymptomatic Mpox Virus Infection in Subjects Presenting for MVA-BN Vaccine

**DOI:** 10.1093/cid/ciad414

**Published:** 2023-07-07

**Authors:** Giulia Matusali, Valentina Mazzotta, Pierluca Piselli, Aurora Bettini, Francesca Colavita, Sabrina Coen, Francesco Vaia, Enrico Girardi, Andrea Antinori, Fabrizio Maggi

**Affiliations:** Laboratory of Virology, National Institute for Infectious Diseases Lazzaro Spallanzani IRCCS (Istituto di Ricovero e Cura a carattere Scientifico), Rome, Italy; HIV/AIDS Unit, National Institute for Infectious Diseases Lazzaro Spallanzani IRCCS (Istituto di Ricovero e Cura a carattere Scientifico), Rome, Italy; Epidemiology Unit, National Institute for Infectious Diseases Lazzaro Spallanzani IRCCS (Istituto di Ricovero e Cura a carattere Scientifico), Rome, Italy; Laboratory of Virology, National Institute for Infectious Diseases Lazzaro Spallanzani IRCCS (Istituto di Ricovero e Cura a carattere Scientifico), Rome, Italy; Laboratory of Virology, National Institute for Infectious Diseases Lazzaro Spallanzani IRCCS (Istituto di Ricovero e Cura a carattere Scientifico), Rome, Italy; Laboratory of Virology, National Institute for Infectious Diseases Lazzaro Spallanzani IRCCS (Istituto di Ricovero e Cura a carattere Scientifico), Rome, Italy; General Direction, National Institute for Infectious Diseases Lazzaro Spallanzani IRCCS (Istituto di Ricovero e Cura a carattere Scientifico), Rome, Italy; Scientific Direction, National Institute for Infectious Diseases Lazzaro Spallanzani, IRCCS (Istituto di Ricovero e Cura a carattere Scientifico), Rome, Italy; HIV/AIDS Unit, National Institute for Infectious Diseases Lazzaro Spallanzani IRCCS (Istituto di Ricovero e Cura a carattere Scientifico), Rome, Italy; Laboratory of Virology, National Institute for Infectious Diseases Lazzaro Spallanzani IRCCS (Istituto di Ricovero e Cura a carattere Scientifico), Rome, Italy

To the Editor—Recent reports in this journal [1] and others [2–5] assessed the prevalence of undiagnosed mpox virus (MPXV) infection either by retrospective testing of clinical specimens for viral DNA and/or presence of antiviral antibodies. These studies contribute to understanding the role of asymptomatic infection in the spread of MPXV during epidemic and interepidemic phases. One of the tools for dealing with the epidemic was the launch of the vaccination campaign with the third-generation MVA-BN vaccine. In Italy, the vaccination program started on 8 August 2022 as preexposure prophylaxis and, considering the epidemic scenario and the limited availability of doses, was targeted to high-risk categories such as laboratory personnel with possible direct exposure to orthopoxviruses (OPXV) and men who have sex with men who met sexual habit–associated risk criteria (ie, multiple sexual partners, recent sexually transmitted infection, chemsex) [6]. Individuals with reported MPXV exposure or previous MPXV infection were considered not eligible. Among the 1549 individuals who received MVA-BN at our institute between 8 August and 9 September, 1244 (80.3%) were naive to vaccinia virus (VACV)–based vaccines. We analyzed serum samples from 141 (11.3%) randomly selected VACV vaccine–naive individuals before the vaccine administration. Samples were tested for anti-MPXV immunoglobulin G (IgG) and neutralizing antibodies (NAbs) by immunofluorescence and plaque reduction neutralization tests, respectively [7]. All were men, the median age was 38 years (interquartile range, 31–43 years), and 70 (49.6%) were people with human immunodeficiency virus (HIV). Overall, 123 serum samples (87.2%) tested negative by both assays, and 18 (12.8%) revealed anti-MPXV IgG at different levels. Importantly, 11 of 18 (61.1%) anti-IgG–positive samples had a concomitant presence of NAbs (Table 1). To investigate whether anti-MPXV antibodies resulted from recent exposure to the virus, we tested the samples for the presence of anti-MPXV immunoglobulin M (IgM) and immunoglobulin A (IgA), serological markers associated with the acute and postacute phases of infection [7–9]. Anti-MPXV IgA and IgM were detected in 8 and 4 samples, respectively, with the serum from individual 17 showing high titers of these early markers. The presence of serum MPXV DNA at a low level was only revealed in individual 18 (cycle threshold, 36.01). Viral DNA detection was not performed on non-blood-derived specimens since they were not collected at the time of vaccination.

**Table 1.** Detection of Anti–*Monkeypox virus* Antibodies

Individual	IgG	IgM	IgA	NAbs
1	1:20	und	und	und
2	1:20	und	und	und
3	1:20	und	und	und
4	1:20	und	und	und
5	1:20	und	und	und
6	1:40	und	und	und
7	1:40	und	und	und
8	1:40	und	und	1:10
9	1:160	und	und	1:20
10	1:80	und	1:20	1:40
11	1:160	und	1:20	1:80
12	1:40	und	1:20	1:10
13	1:80	und	1:20	1:20
14	1:160	und	1:20	1:80
15	1:160	1:20	und	1:80
16	1:80	1:20	1:20	1:160
17	1:80	1:320	1:80	1:80
18	1:320	1:40	1:20	1:20

Abbreviations: IgA, immunoglobulin A; IgG, immunoglobulin G; IgM, immunoglobulin M; NAbs, neutralizing antibodies; und, undetected.

Although the only detection of anti-MPXV IgG and NAbs cannot exclude previous exposure to other OPXV due to serological cross-reactivity, the simultaneous presence of IgA and/or IgM suggests a recent OPXV infection. Individuals 16, 17, and 18, who showed the widest range of antibody positivity, were antiretroviral therapy–suppressed aviremic people with HIV with a CD4 count >500 cells/μL; 2 of them (individuals 16 and 18) reported sexually transmitted infections in the last year. More importantly, the presence of MPXV DNA in the serum of subject 18 confirms recent MPXV infection at the time of the first MVA-BN dose, as viremia is usually described in the first 3 weeks from symptom onset [10]. In conclusion, our findings suggest the importance of monitoring people at higher risk of infection for possible cases of asymptomatic MPXV. Even if different studies report a low to relevant impact of unrecognized and asymptomatic MPXV infections [1–5], there is consensus on the need for prevention and control measures that encompass enhanced surveillance, wider availability of testing, access to vaccination, and adequate risk communication strategies.

## Notes

***Author Contributions.*** G. M. performed data analysis, designed the experimental study, and wrote the manuscript. V. M. and A. A. conceived and wrote the vaccination protocol, organized the sample and data collection, and obtained the funding. P. P. was responsible for data management. F. C. analyzed data. A. B. and S. C. performed experiments. F. M. supervised the experimental work and edited the manuscript.

***Patient consent.*** All of the participants included in the study provided written informed consent. The study was approved by the Ethical Committee of the National Institute for Infectious Diseases Lazzaro Spallanzani (approval number 41z/2022).

***Financial support.*** The work was supported by the Ministero della Salute, Italy, Ricerca Corrente Linea 1.

***Potential conflicts of interest.*** E. G. reports unrestricted grants from Gilead Sciences and Mylan to National Institute for Infectious Diseases stays for the Istituto Nazionale per le Malattie Infettive, and payment or honoraria for educational presentations from Gilead Sciences. All other authors report no potential conflicts.
